# Remarkable enhancement of thermal stability of epoxy resin through the incorporation of mesoporous silica micro-filler

**DOI:** 10.1016/j.heliyon.2021.e05959

**Published:** 2021-01-18

**Authors:** Farzana Yeasmin, Abul K. Mallik, Adib H. Chisty, Fataha N. Robel, Md. Shahruzzaman, Papia Haque, Mohammed Mizanur Rahman, Nanami Hano, Makoto Takafuji, Hirotaka Ihara

**Affiliations:** a Department of Applied Chemistry and Chemical Engineering, Faculty of Engineering and Technology, University of Dhaka, Dhaka, 1000, Bangladesh; b Department of Applied Chemistry and Chemical Engineering, Noakhali Science and Technology University, Sonapur, Noakhali, 3814, Bangladesh; c Department of Applied Chemistry and Biochemistry, Faculty of Engineering, Kumamoto University, 2-39-1 Kurokami, Kumamoto, 860-8555, Japan

**Keywords:** Epoxy, Glass transition temperature, Thermal stability

## Abstract

For the first time, we incorporated mesoporous micro-silica (5 μm, pore size = 50 nm) as a filler in epoxy resin aiming to enter polymer into the pore of the silica. As expected, the thermal stability of the composite increased remarkably, followed by noteworthy thermal degradation kinetics when compared to the controlled cured epoxy resin. Composites were prepared by the direct dispersion of modified nano-silica, modified mesoporous micro-silica, unmodified mesoporous micro-silica, non-porous micro-silica, and irregular micro-silica of various pore sizes as fillers in diglycidyl ether of bisphenol-A epoxy resin via ultra-sonication and shear mixing, followed by oven-curing with 4,4-diaminodiphenyl sulfone. DSC and TGA analyses demonstrated a higher glass transition temperature (increased by 3.65–5.75 °C) and very high activation energy for thermal degradation (average increase = 46.2%) was obtained for the same unmodified silica composite compared to pure epoxy, respectively.

## 1 Introduction

Epoxy is an important class of thermosetting polymers that has stimulated considerable research interest since its invention in 1938 owing to its versatility in industrial applications, such as raw materials of surface coatings, adhesives, circuit boards, insulators, packaging matrices and encapsulants of electrical appliances and matrix resin for high-performance fiber-reinforced composites in the transportation sector etc. [1, 2]. Several unique properties of cured epoxy resin, such as excellent mechanical and chemical resistance, superior adhesion strength, enhanced durability, extended flame retardancy etc. are achieved due to instability of epoxide rings via reaction with aliphatic or aromatic amines, polyamides, polycarboxylic acids, polyphenols, polysulfides, and anhydrides [3, 4]. For any specific application, the desired range of the properties of epoxy-based composites is strongly related to the selection of an appropriate epoxy-hardener system involving suitable process variables (time and temperature of curing), stoichiometric ratio, degree of cross-linking and choice of reinforcement filler used [1, 5]. Industrial applications, often at high temperature, usually demand good adhesion and mechanical properties, rough service conditions, and load-bearing properties, along with erosion and corrosion resistance. In these applications, e.g., electrical devices and electronic and aerospace formulations, optimal results are obtained with epoxy composites cured by sterically hindered aromatic amine, owing to their densely cross-linked structure along with enhanced thermal stability [3, 6]. Despite having such superior properties, cross-linked resin needs improvement in fracture toughness and thermal stability, which would enhance its dimensional stability and a reduction in brittleness to prolong its service life; these features are being addressed by researchers.

The largest volume of commercially available epoxy, considered as the lifeblood of the epoxy field, is diglycidyl ether of bisphenol-A (DGEBA), which has demonstrated noteworthy thermal properties when cured with an aromatic amine-based hardener such as diamino diphenyl sulfone (DDS). Silica is a negatively charged popular oxide filler for thermosetting polymers with better thermal properties [7]. Studies by Ma et al. and Gurung et al. revealed significant improvement in the thermo-mechanical properties of resin cured with 4, 4′-DDS and reinforced by silica filler [3].

**Figure 1:**
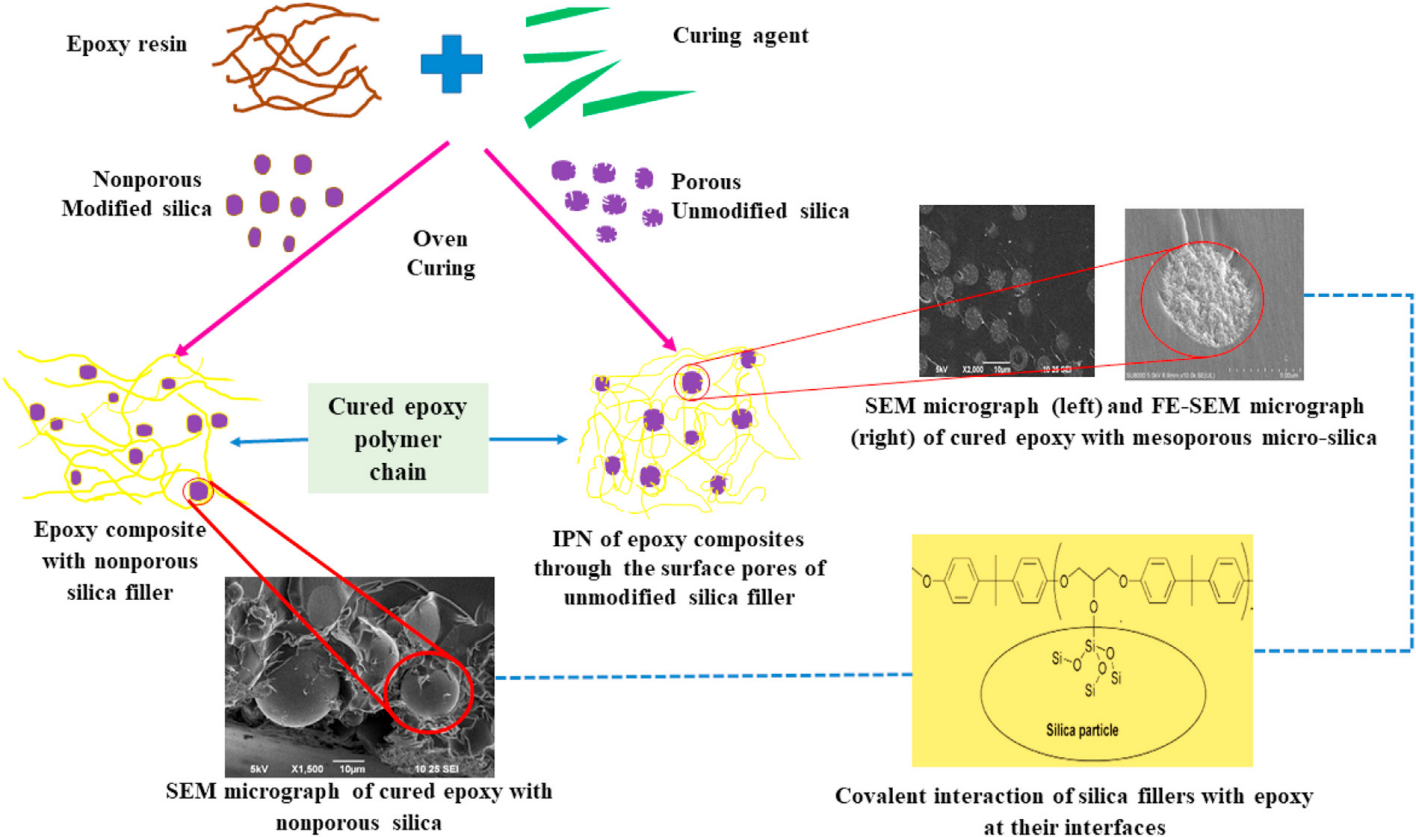
Schematic illustration of formation of epoxy polymeric resin composite through incorporation of nano/micro silica filler.

There are many studies that have reported silica filler particles acting as a physical cross-linker in epoxy resin, in which a relatively small volume of well-dispersed silica nano-/micro-particles can induce enhanced stiffness, improved thermal stability, better electrical insulation, reduced shrinkage upon curing, and decreased thermal expansion coefficients and fulfill the required mechanical properties for composites [8, 9, 10]. Owing to the Si–OH groups on its surface, silica particles show reactivity toward the epoxide ring-opening reaction, forming an Si–O–C linkage with restricted molecular mobility of resin, better adhesion, and increased interface area without the presence of any other catalyst or promoter [11, 12, 13]. Although a comparison between nano- and micro-silica fillers regarding the thermal properties of composites were reported [14, 15], but no clear evidence of the efficacy of mesoporous micro-silica over that of nano-silica fillers for the properties of epoxy resin has yet been obtained. Moreover, the mesoporous silica-based composites are relatively newer materials having many unrevealed properties, which have received attentions of the researchers lately; Recent studies ascertained that many commercially available nanoscale silica fillers could be suspended homogeneously in organic solvent and showed strong reactivity towards epoxy resins simultaneously without addition of any catalyst [9]. Improving the bonding between the resin matrix and porous filler particles by forcing the mechanical interlocking of the resin polymer inside the filler pores was initially suggested by Bowen and Reed [16, 17]. Owing to an ordered structure without any hydrolysable silane coupling agents, a high surface-area-to-volume ratio and simple functionalization of the nanopores, mesoporous silica microfillers may provide the inclusion of macromolecules in the nanopores with intimate interactions between the polymer and inorganic phase, ultimately resulting in an interlaced polymer network with (as exemplified by Figure 1) synergistic effects on its properties when compared with nonporous nano-fillers [18, 19, 20]. This interweaved polymer network cannot be pulled apart without any bond cleavage and hence provide higher thermal stability [21].

In case of preparation of mesoporous silica based polymer composites, direct mixing of the mesoporous silica into the polymer via melt blending or solution blending is the simplest method. Melt blending is efficiently operable only when the mesoporous silica is assorted into the polymer above the melting point or above the glass-transition temperature (T_g_) of polymers. This temperature issue can be avoided simply by mixing mesoporous silica and polymer in any suitable organic solvent following solution blending which can induce a satisfactory mixing at molecular level and is relatively cheap [22]. So, in present study, we followed the solution blending polymerization technique, where ethanol appeared as an appropriate solvent to uniformly disperse silica fillers into the epoxy matrix as illustrated in the SEM micrographs. The solvent used was further removed successfully from the composite as evident from the TGA data.

Prevention of thermal degradation during the shelf lifetime of thermosetting composites involving complex degradation can be assessed by the study of thermal degradation kinetics, which provides valuable information to optimize the processing conditions or service life of the composites [45]. To the best of our knowledge, the thermal degradation kinetics of amine cured epoxy-silica composites have not been reported yet in literature. So, in present study, we followed the model-free Flynn-Wall-Ozawa Method to investigate kinetic analysis of thermal degradation of the epoxy composites which provides a simple relationship between the conversion dependent activation energy, heating rates and isoconversion temperature and calculated the activation energy and pre-exponential factors of the composites as a function of thermal degradation ranging from 5-90 %.

Herein, we aimed to observe the effect of pore and pore size of unmodified mesoporous micro-silica on the interactions between the filler and the matrix. We compared these effects with those for modified nano-silica, unmodified nonporous micro-silica and modified porous micro-silica. We also considered the enhanced thermal properties of these composites in terms of thermal stability, glass transition temperature, and the thermal degradation kinetics of 4,4′-DDS cured DGEBA epoxy resin, as demonstrated in Figure 1. As expected, epoxy composites with small amounts of unmodified mesoporous silica, as low as 3 wt. %, revealed remarkably better thermal performance (with increasing pore size of mesoporous silica) than composites containing modified nano-/micro-silica and controlled epoxy, signifying improved thermal barrier towards degradation induced by nanopores of the unmodified silica fillers into the matrix with increased entanglement network which can also be correlated with the SEM micrographs. It is worth mentioning that the kinetic energy for thermal degradation of the mesoporous silica-based composites had promisingly increased up to a maximum of 46.2% increment (for composites with 50 nm pore size micro silica fillers). A view of the filler–matrix interface demonstrates that the nanopores of the micro-silica moieties were able to accommodate the cured polymeric chains, comprising an intricate composite architecture, which ultimately resulted in a pronounced thermal stability.

## 2 Experimental

### 2.1 Materials and methods

DGEBA epoxy resin (molecular weight 340 g/mol, epoxied equivalent weight of 173 g/eq) and 4,4′-DDS (molecular weight 248 g/mol, amine equivalent weight of 62 g/eq) serving as a cross-linker were purchased from Sigma-Aldrich, USA. Mesoporous silicas (5 μm) were used as inorganic reinforcement (Table 1) and obtained generously as gift from AGC Si-Tech Co., Ltd. Tokyo, Japan. Other silica particles were purchased from Wako, Osaka, Japan. Mercaptopropyl trimethoxysilane (MPTS) was purchased from TCI, Tokyo, Japan.

**Table 1:** Variation of silica particles used.

No.	Type	Particle size	Pore size (nm)	Modifier
1	Non-porous, hydrophobic	10 nm	None	MPTS∗
2	Meso-porous, hydrophobic	3μm	10	MPTS∗
3	Meso-porous, hydrophilic	5 μm	20	None
4	Meso-porous, hydrophilic	5 μm	30	None
5	Meso-porous, hydrophilic	5 μm	50	None
6	Non-porous, hydrophilic	20 μm	None	None
7	Meso-porous, hydrophilic	63–212 μm, irregular size	50	None

∗MPTS = Mercaptopropyl trimethoxysilane

### 2.2 Fabrication of the epoxy-silica composites

DGEBA-based epoxy composites filled with 3 wt. % filler of various types of silica were prepared by a solvent-assisted dispersion procedure, followed by a solution-blending polymerization technique, based on previous studies [23, 24].

Initially, different types of preweighted silica nano-/micro-particles were dispersed in ethanol at room temperature by ultrasonic treatment until a milky white dispersion appeared. The desired amount of resin, preheated at 50 °C, was then mixed with the dispersed silica. The mixture was further sonicated to disperse the silica particles uniformly and heated at 80 °C on a hot plate to evaporate the solvent. Next, 35 phr of 4,4′-DDS, dissolved in acetone, was added to the mixture, followed by a high-shear mixer treatment and degassing, ultimately resulting in a homogeneously mixed, bubble-free liquid mixture. The final mixture was poured into a preheated glass mold and placed in a muffle furnace, following isothermal curing at 110 °C for 2 h and post-curing at 200 °C for 4 h, respectively.

Seven different samples reinforced with seven different types of silica were prepared and denoted as ENC-X and EMC-XpY or EMC-w for epoxy-silica nano-composite and epoxy-silica micro-composites, respectively (where X indicates the particle size of the silica filler, pY indicates the pore size of the mesoporous silica, and w stands for wakogel), represented in Table 2. Another sample of neat epoxy resin was cured following the described methodology using no silica, simultaneously with the test samples to be used as reference (denoted as CE, control epoxy).

**Table 2:** Nomenclature of the fabricated composites.

No. of composite	Category of Silica			Nomenclature
	Particle size	Pore size, nm	Modification by MPTS	
1	No silica			CE
2	50 nm	-	Modified	ENC-50
3	3 μm	10	Modified	EMC-3p10
4	5 μm	20	Unmodified	EMC-5p20
5	5 μm	30	Unmodified	EMC-5 p30
6	5 μm	50	Unmodified	EMC-5 p50
7	20 μm	-	Unmodified	EMC-20
8	63–212 μm	50	Unmodified	EMC-w

### 2.3 Characterization techniques

The attenuated total reflectance (ATR) and FT-IR spectrum of the prepared samples were recorded using an FT-IR spectrometer (Irprestige-21 model, Shimadzu Corporation, Japan) equipped with an ATR device in transmittance mode. Elemental analysis of modified silica nano-and micro-fillers was accomplished via a Yanaco CHN Corder MT-6 apparatus (YANACO Co., Ltd., Kyoto, Japan).

The microscopic morphology of the fracture surface of the composites was obtained through scanning electron microscopy (SEM) using a JEOL 6400 microscope at an accelerating voltage of 5 kV. The cross-sectional view of the sample specimen was assessed after sputter coating with osmium. The sample exhibiting greater improvement in thermal properties was further analyzed by FE-SEM (Hitachi SU-8000, JAPAN) for better understanding and confirmation of the interaction behavior of the filler particles with the matrix at their interfaces. Differential scanning calorimetry (DSC) (Seiko EXTRA6000 with a DSC6200 instrument) experiments were carried out within a temperature range of 30–330 °C at a heating rate of 2 °C/min. The fabricated composites of weight 5 ± 2 mg were heated in an aluminum pan, under an N_2_ atmosphere. TGA analysis of the samples was performed by a TGA-50H instrument (Shimadzu Corporation, Japan). The sample specimen with a weight of 5 ± 2 mg was placed in an alumina dish, and the temperature was raised from 25 to 800 °C at various heating rates (5 °C/min, 10 °C/min, 15 °C/min, and 20 °C/min) under N_2_ atmosphere by isothermal scans. The thermal stability of the samples was monitored as a function of temperature with a heating rate of 10 °C/min. The decomposition temperature, T_d_, was considered at 10% weight loss, and the char residue was compared at 600 °C. The kinetics of the thermal degradation in terms of activation energy (E_a_) of the samples was evaluated using the TGA data at 5 °C/min, 10 °C/min, 15 °C/min, and 20 °C/min through the *FLYNN-WALL-OZAWA* (FWO) method.

### 2.4 Analysis of thermal degradation kinetic

#### 2.4.1 Theoretical approach

For any thermogravimetric analysis, the rate of degradation reaction (X) can be defined as

(1) X = Actual weight loss/Total weight loss = (W_0_-W_t_) / (W_0_-W_f_)

where, W_0_ = initial weight of the sample, W_t_ = actual weight of the sample, W_f_ = final weight of the sample and X = degree of decomposition [25].

The majority of kinetic methods practiced in thermal analysis consider the degradation rate to be a function of variables, T (temperature) and X (weight loss of the sample). Using Eq. (1), the decomposition rate (dX/dt) can be expressed as a function of X, as follows [25],

(2) dX/dt = Kf(X)

K is the Rate constant and f(X) is the differential expression for a kinetic model function, which provides information regarding the mechanism of reactions.

The dependence of temperature on process rate is typically parameterized through Arrhenius equation:

(3) $$\text{K}\left(\text{T}\right)\;=\text{ A}e^{-\frac{E}{RT}}$$

where, A stands for the pre-exponential factor, R is the Universal Gas Constant (8.314 Jk^−1^ mol^−1^), T is the absolute temperature (273.15 K) and E denotes the Activation Energy of the degradation reaction (KJ/mol), respectively [19, 59].

By combining Eqs. (2) and (3), the kinetics of solid-state reaction can be described by the following rate equation obtained [25,59].

(4) $$\text{dX/dt }=\text{ A}e^{-\frac{E}{RT}}\text{f}\left(\text{X}\right)$$

In case of TGA analysis, the temperature of the sample is changed linearly with time [19, 59], i. e., the constant heating rate,

(5) ß=dT/dt

Combining Eqs. (4) and (5)

(6) $$\text{dX/dT }=\text{ A/ß}e^{-\frac{E}{RT}}\text{f}\left(\text{X}\right)$$

Therefore, Eq. (6) is the basic equation to define the kinetic parameters based on the TGA data in order to calculate the apparent activation energy, depending on the degree of conversion (X) and the heating rate (ß) [19, 59].

Numerous studies have followed several methods like Flynn-Wall-Ozawa method (FWO), Kissinger-Akahira-Sunose method (KAS), Phadnis-Deshpande method, Van Krevelen method, Prout-Tompkins autocatalytic model, Coats-Redfern method etc. to determine the apparent activation energy based on TGA data at one or different heating rates whereas the calculated kinetic parameters strongly depend on the method of calculation [24, 26]. Since, Ozawa's method does not necessitate the knowledge of the reaction mechanism [27], the present study utilizes Ozawa's method to investigate the thermal degradation kinetics parameters, apparent activation energy and pre-exponential factor of thermal degradation at various conversions of epoxy-silica nano/micro-composites assuming the degradation mechanism following first order kinetics.

### 2.5 Flynn-Wall-Ozawa Method

Model free isoconversional methods are usually employed to determine the activation energy (which remains constant throughout the whole reaction mechanism) as such methods can provide the evaluation of both the simple and complex chemical reactions. A well-fitting isoconversional integral method, which shows a good mathematical compliance with the values obtained from the phenomenological reaction models as well as the Kissinger method. Based on the complicated degradation reaction mechanism of the cured epoxy resin, the data for activation energy of degradation obtained from Flynn-Wall-Ozawa (FWO) method is considered to be more reliable as the method allows to provide the data of activation energy at all the points of degradation in TGA curves.

This method is based on the fact that, the conversion function f(X) does not change with the alteration of the heating rates for all values of X. So, the temperature corresponding to fixed values of X is measured from the experiments at different heating rates ß.

The integration form of Eq. (6) from an initial temperature T_0_, corresponding to a degree of conversion X_0_, to the peak temperature T_p_, where X = X_p_, provides

(7) $$\text{g}\left(\text{X}\right)=\overset{Xp}{\underset{Xo}{\int }}\frac{dX}{f\left(X\right)}=\text{A/ß}\overset{Tp\;}{\underset{To}{\int }}\exp\left(-E/RT\right)dT$$

here, g (X) implies the integral function of conversion.

Let, x = E/RT, then from Eq. (7), we can write:

$$\text{A/ß}\overset{Tp\;}{\underset{To}{\int }}\exp\left(-E/RT\right)dT=\left(\text{AE}\right)\text{/}\left(\text{ßR}\right)\text{×p}\left(\text{x}\right)\text{,}$$

Thus, g(X) can be expressed more conveniently as:

(8) g (x)=(AE)/(ßR)×p(x)

Taking logarithms on both sides of Eq. (8),

(9) $$\text{logß}=\log\frac{\text{AE}}{\text{g }\left(\text{x}\right)\text{R}}+\log\text{p}\left(\text{x}\right)$$

From Doyle's approximation i. e. when x≥20 the function p(x) can be adopted to the following approximation:

$$\text{p}\left(\text{x}\right)≅0.0048e^{-1.0516X}$$

(10) logp(x)≅−2.315−0.4567X

By numerical integration, the expression for logp(x) can be estimated using the trapezoid rule with a step of 10^−3^ with respect to the temperature. That is, from equation no (9) and (10), we find:

(11) $$\text{logß}=\log\frac{\text{AE}}{\text{g }\left(\text{x}\right)\text{R}}-2.315-0.4567\frac{E}{RT}$$

here, A and R are constant and for any particular conversion, g (X) is a constant [59].

All the equations from (7) to (10) were thoroughly derived to obtain Eq. (11) which is well known as Flynn-Wall-Ozawa method [19, 27, 59]; used to determine the activation energy without any prior knowledge of the reaction mechanism from the linear dependence of logß vs 1000/T at different heating rates (5, 10, 15, 20 °C/min were used in this study). The activation energy for degradation reaction, E is calculated from the slope of such straight line being equal to -0.4567 (E/R) and the intercept of that very straight line provides the value of A, the pre-exponential factor (min^−1^).

## 3 Results and discussion

### 3.1 Chemical structure analysis by ATR-FTIR

**Figure 2:**
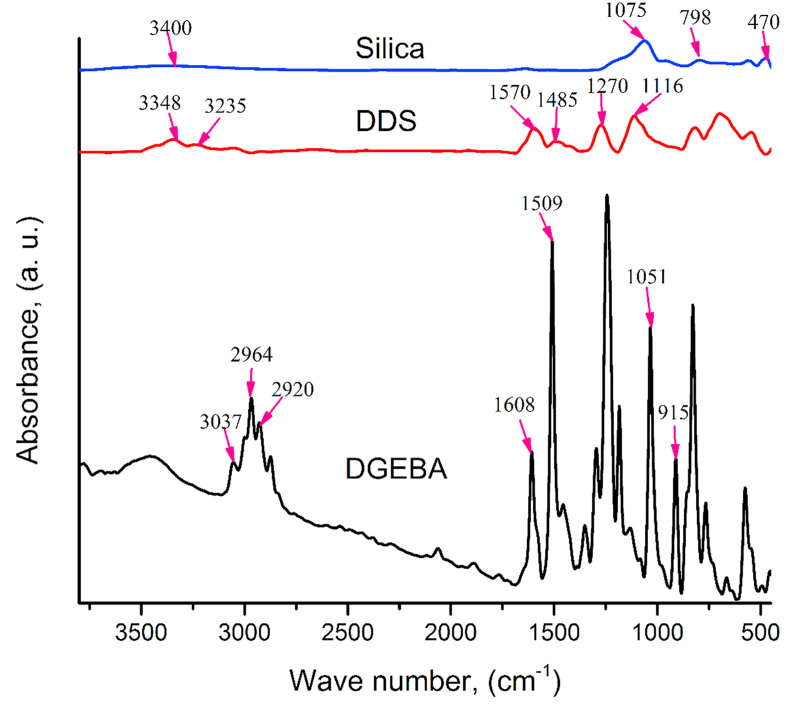
FT-IR spectra of pristine epoxy resin (DGEBA), hardener (4,4′-DDS) and silica filler.

FT-IR spectral analyses were used to investigate the functional groups of the epoxy resin, 4,4′-DDS hardener, and silica particles, as shown in Figure 2. The FT-IR spectrum of DGEBA showed two characteristic absorption peaks of epoxide ring between 4000 cm^−1^ and 400 cm^−1^. The sharp peak at 915 cm^−1^, the reference peak, is assigned to the C–O deformation of the oxirane ring, while the second band, located at 1051 cm^−1^ represented the C–O–C stretching of the epoxy group and another band at 3037 cm^−1^, is attributed to the C–H stretching of the methylene group in oxirane [28, 29, 30]. Reference peaks at around 1509 and 1608 cm^−1^ correspond to C–C stretching vibration of aromatics and C=C stretching vibration of aromatics, 2920 and 2964 cm^−1^ are related to the C–H stretching vibration of –CH_2_- and C–H stretching vibration of –CH_3_, respectively [29, 30].

4,4′-DDS as a hardener showed the most remarkable peaks at 1116 cm^−1^ and 1270 cm^−1^, ascribed to the symmetric and asymmetric stretching of –SO_2_-, respectively. In addition to symmetric and asymmetric stretching of –SO_2_-, strong peaks at 1450–1600 cm^−1^ and 3200–3400 cm^−1^ correspond to the bending vibrations of –NH– and stretching of NH_2_- (symmetric and asymmetric) in DDS, respectively [31, 32]. The FT-IR spectrum of unmodified silica particles also demonstrated clear peaks at 1075 cm^−1^,798 cm^−1^and 470 cm^−1^ responsible for the asymmetric, symmetric as well as bending modes of SiO_2_ along with peak at 3400 cm^−1^, due to the –OH stretching of silanol groups [33, 34, 35].

Due to the strong and broad peaks of Si–O (950-1100 cm-^1^) and –OH (3000-3780 cm-^1^) of silanol group, the silane functional groups are not easy to detect by IR spectrum [36]. The surface modification of the silica particles was confirmed by elemental analysis, and the obtained results are shown in Table 3.

**Table 3:** Elemental analysis data of nano and micro fillers modified by MPTS.

Silica	C (%)	H (%)	N (%)
50 nm silica particle	1.60	0.43	0
3 μm silica particle	2.26	1.05	0

**Figure 3:**
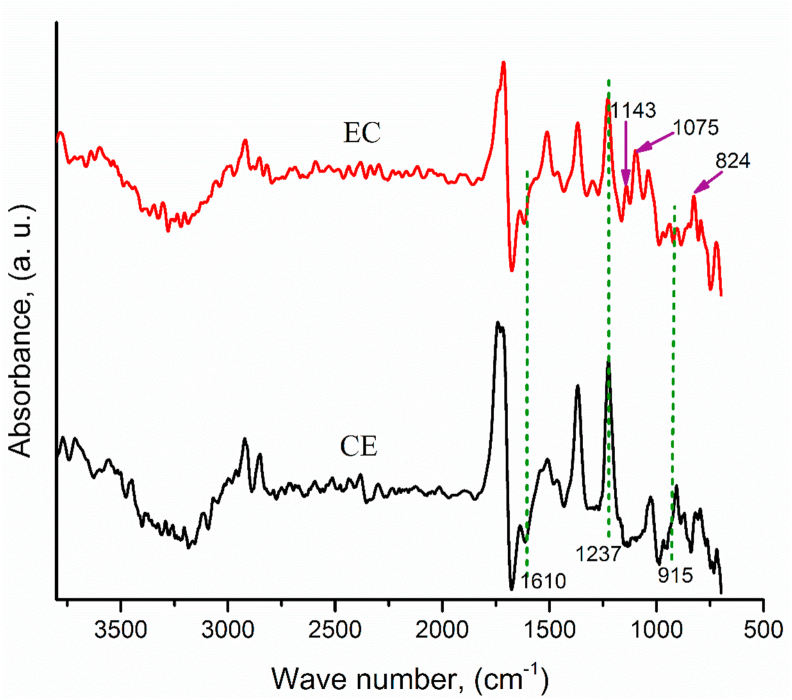
ATR-IR spectra of control epoxy (CE) and epoxy composite (EC).

The ATR-IR spectra of the composite samples revealed the extent of curing of the epoxy resin, as depicted in Figure 3. The prominent features were the decreasing and almost complete disappearance of the epoxy ring at 915 cm^−1^ and the N–H stretching at 1610cm^−1^ confirming the complete ring opening reaction of the epoxy resin through the cross-linking of the end epoxy groups with the hardener in the curing process resulting a three-dimensional crosslinked network [37]. The presence of peak absorption at 1237 cm^−1^ was due to stretching of C–N formed by cross-linking of epoxy ring with amine group hardener [1, 21]. In the spectra of epoxy-silica composites, peaks at 824 cm^−1^, 1075 cm^−1^, and 1143 cm^−1^ are attributed to the presence of Si–OH bending, Si–O–Si stretching vibrations of silica filler, and the Si–O–C bond due to the covalent bonding of silica fillers with the matrix, respectively, confirming the inclusion of silica fillers, which was absent in the CE [33, 38, 39].

### 3.2 Morphological study

**Figure 4:**
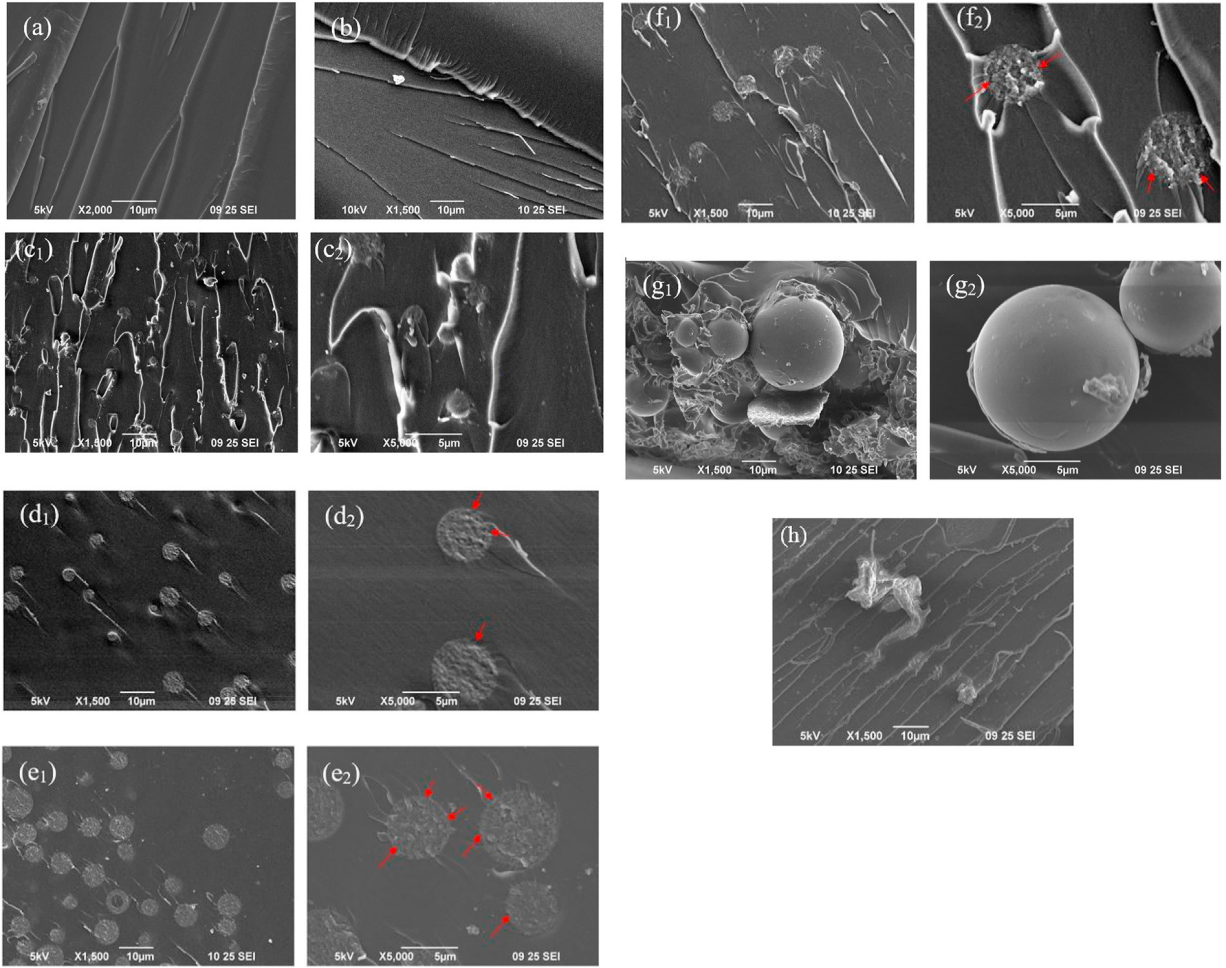
SEM micrographs of the fracture surface of the epoxy composites: (a) CE, (b) ENC-50, (c) EMC-3p10 at low (i) and high (ii) resolution respectively, (d) EMC-5p20 at low (i) and high (ii) resolution respectively, (e) EMC-5p30 at low (i) and high (ii) resolution respectively, (f) EMC-5p50 at low (i) and high (ii) resolution respectively, (g) EMC-20 at low (i) and high (ii) resolution respectively, (h) EMC-w.

The SEM micrographs of the fractured microstructure of the fabricated samples demonstrate the difference in the interfacial interaction between the matrix and silica fillers and the morphology-structural property correlations of the composites containing various sizes of silica fillers. In case of the CE, the river line path of the smooth fracture surface of Figure 4(a) depicts the evolution of the damage and energy dissipation during cracking; meanwhile, in Figure 4(b), the fractured surface was almost similar to that of Figure 4(a), as 50 nm fillers were hard to detect using SEM. However, the augmented roughness of the fractured surfaces of Figure 4(b), when compared with the smooth surface of the CE, only confirmed the insertion of silica fillers in the epoxy matrix, even though the dispersion and compatibility of the nano-silica filler with the resin matrix could not be understood [40]. The micrographs showed in Figure 4(c)–(f) demonstrated satisfactory homogeneous dispersion of silica in the matrix, implying good miscibility between the silica particles and cured epoxy matrix along with the absence of voids surrounding the silica particles, unlike in Figure 4(g), where it would be precise to mention that all the samples were prepared following identical conditions.

**Figure 5:**
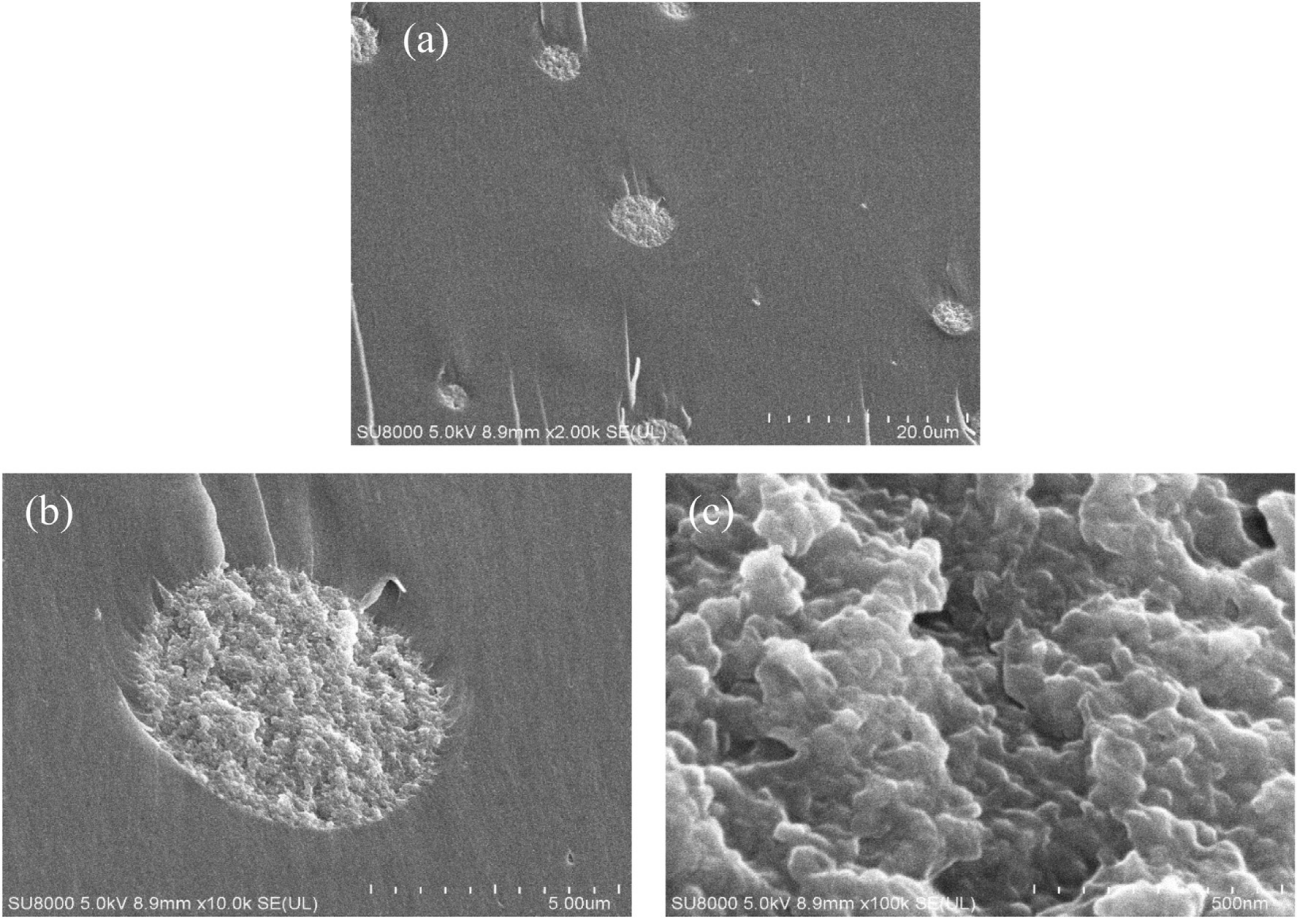
FE**-**SEM micrographs of the fractured surface of the epoxy composite EMC-5p50 containing 5μm silica of pore 50 nm with higher magnification: (a) of 20 μm, (b) of 5 μm and (c) of 500 nm.

By observing both the lower and higher magnification micrographs for epoxy composites with mesoporous micro-silica, it was concluded that the well-dispersed modified mesoporous silica particles (Figure 4(c_1_) and (c_2_)) were embedded in the epoxy matrix, showing good adhesion at the interfaces with no debonded particles. Unmodified mesoporous silica particles, shown in Figure 4(d_1_), (e_1_), and (f_1_), exhibited smoother surface morphologies compared with the other micrographs and excellent matrix-filler interfacial interaction with a similar homogeneous network throughout the cured matrix, more clearly understood at higher magnification in Figure 4(d_2_), (e_2_), and (f_2_). The strong interfacial adhesion between the matrix and silica filler might be due to the reaction of the epoxy groups with Si–OH groups present on the filler surface, demonstrating no evidence of particle-matrix debonding or plastic void growth [33, 41]. FE-SEM micrographs of the composites with 50 nm porous micro-silica were further observed to focus more accurately on the matrix filler interfacial interaction nature, as shown in Figure 5, and analogous observations with SEM micrographs were recognized. This strong interface adhesion might pertain to the association of the ultra-large interfacial area per volume of unmodified mesoporous micro-silica, penetrating the polymer molecules into the surface pores of the silica, which eventually leads to the formation of an interpenetrating network (IPN), as indicated by the red arrows in the micrographs. The mesoporous silica would act as sites for nucleation, with their uniform dispersion in the matrix facilitating the significant enhancement of the entangled network in the cured epoxy composites. Such significant compatibility associated with the composites EMC-5p20, EMC-5p30, and EMC-5p50 reveals the major impact of the epoxy micro-composites with unmodified mesoporous micro-silica fillers on the enhanced thermal properties, as compared with the composites with modified nano-silica [42]. From Figure 4(g_1_), in the sample with 20 μm nonporous silica filler, small agglomerates are evident, which clearly implies that the ultrasonic and shear treatments for the dispersion and mixing of the silica fillers with resin matrix were insufficient; meanwhile, the distinct debonding between the fillers and matrix, as shown in Figure 4(g_2_)_,_ clearly confirms the deterioration of the particle-matrix adhesion strength due to generation of cavities or voids with no particles [43]. In Figure 4(h), a smooth composite surface with slight roughness at a distinct location reveals the nonuniform distribution of the fillers in the matrix due to the variation in size at the micrometer scale.

### 3.3 Thermal properties analyses

#### 3.3.1 Differential scanning calorimetry (DSC) analysis

**Figure 6:**
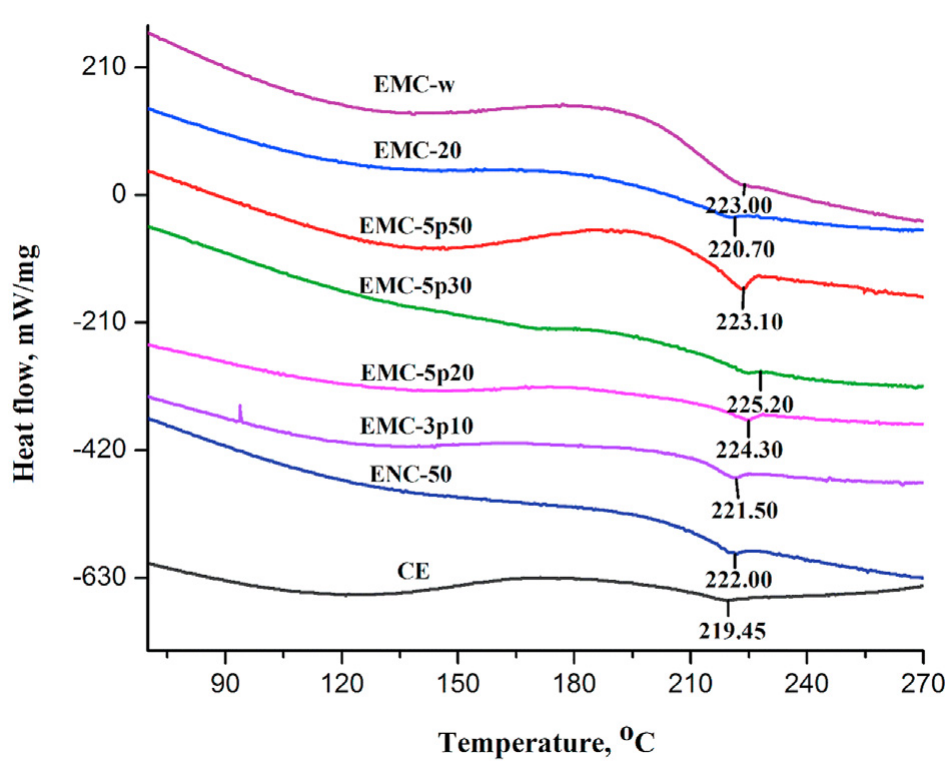
DSC thermogram of control epoxy resin and epoxy-silica nano/micro-composites.

DSC is a practical and reliable approach to evaluate the thermal stability, phase transition temperature, and glass transition temperature (*T*_g_) of polymer and other materials [44]. The *T*_g_ of the materials appraises the thermal deformation property of materials in real service life [45]. DSC analyses of all the cured epoxy composites, presented in the thermogram of Figure 6, showed similar behavior with augmentation of the *T*_*g*_ of the cured composites with respect to the CE. The main exothermic transition appeared in the temperature range of approximately 150–220 °C, due to the addition reactions between the epoxy ring and amines of the hardener, side reactions such as etherification of hydroxyl groups and epoxy groups, homopolymerization of epoxy ring opening, and thermal decomposition of weak bonds, occurred in the high-temperature region [6, 46]. These transitions became blunt followed by reduced height of the exothermic peak for epoxy composites than that in control epoxy, implying the affluence of initiation of curing reaction in the epoxy composites by silica particles bearing surface –OH groups [47]. The inflection point temperature of this exothermic peak was considered as *T*_g_ [6].

Table 4 included the temperature values of the cured epoxy samples obtained from the DSC curves. All the epoxy-silica systems exhibited an increasing trend for *T*_g,_ as evident from Table 4 and Figure 6. After the *T*_g_ point, another gentle exothermic peak was found for almost all the samples which was may be due to the homopolymerization or etherification of the residual epoxy groups [6]. In case of control epoxy, this exothermic transition was not so mild compared to those of other composites, which ascribed that silica fillers participated in crosslinking with the epoxy resin during the long-time curing steps. The initiation of a faster cross-linking between the silica particle and DGEBA through the intermolecular interaction between the hydroxyl groups on the silica surface and the hydroxyl groups produced from the epoxy rings in curing reactions can drag to hinder the segmental mobility of the polymeric chains [46, 48]. In ENC-50 and EMC-3p10 with lower augmentation of *T*_*g*_, MPTS modified silica particles were used in order to confirm deagglomeration, better dispersion and improved compatibility between the epoxy and coated silica nanoparticles; but these coated fillers ultimately exhibited relatively weak physical interaction (lower degree of cross-linking) toward the resin consistently as reported by Baller and co-workers [49]. EMC-5p20, EMC-5p30, and EMC-5p50 with unmodified mesoporous silica carried a large number of surface –OH groups and showed a higher degree of increase in *T*_g_, inducing the possibilities of assimilating the organic species into their ordered mesoporous structures, ultimately providing an increased intermolecular interaction between the silanol groups on the silica surface and hydroxyl groups in the epoxy matrix [50, 51]. Thus, we can postulate that the composites of the unmodified mesoporous silica with higher increase in *T*_*g*_ contain denser cross-linked morphology providing incremental frictional force to the mobility of polymeric chain, which were coherent with the SEM and FE-SEM micrographs. EMC-w, containing wakogel with 50 nm pores also showed comparable value of *T*_*g*_ which pointed towards the incorporation of higher density of polymeric chain into the open porous structured silica fillers. EMC-20, micro-composites of 20 μm nonporous unmodified silica showed a lower increment in *T*_g_, attributed to the lesser cross-linking density than that of the micro-composites of mesoporous silica, where silica acted as physical crosslinker only. Formation of free volume at the resin–filler interface, as observed by SEM analyses due to the larger size of the unmodified silica particles was responsible for the reduction in the cross-linking density of the EMC-20 composite.

**Table 4:** Temperature results from DSC thermograms of prepared sample composites.

Sample name	T_g_, (°C)
CE	219.45
ENC-50	222.00
EMC-3p10	221.50
EMC-5p20	224.30
EMC-5p30	225.20
EMC-5p50	223.10
EMC-20	220.70
EMC-w	223.00

#### 3.3.2 Thermogravimetric analysis (TGA)

**Figure 7:**
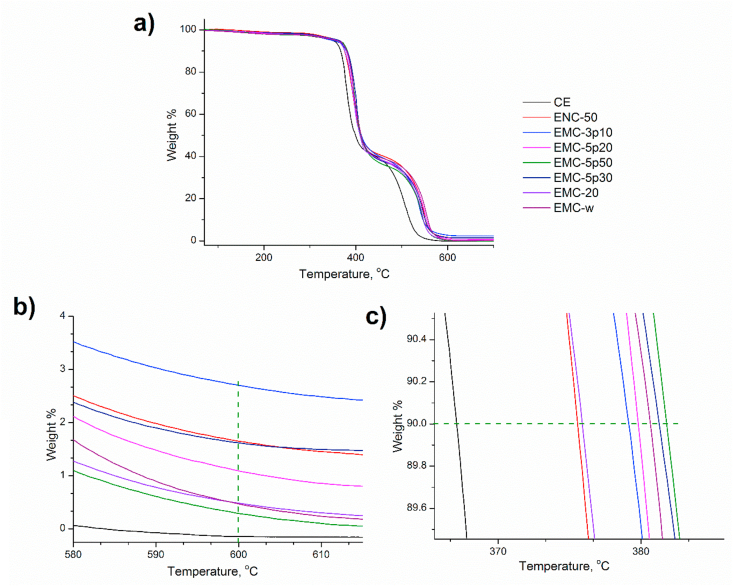
(a) TGA thermogram of 4,4′-DDS cured epoxy-silica composites, (b) close view of 10% degradation, (c) close view of mass residue at 600 °C under N_2_ atmosphere.

TGA thermograms of the samples and analyzed data are shown in Figure 7 and Table 5, respectively. In Figure 7, analogous patterns of thermal degradation of all the samples depicted the deterioration of only organic moieties present in the resin. The successful removal of trace water and solvents was attained during fabrication, as no weight loss was found below 120 °C. At a temperature of approximately 240 °C, degradation started gently through the homolytic fission of chemical bonds in the ester linkage of the network, owing to the dehydration of oxypropylene group, –CH_2_-CH(OH)-, with the subsequent formation of double bonds. Isomerization reactions, chain transfer, intermolecular cyclization and other radical reactions produced during the first stage of degradation resulted in their degradation at the second stage of weight loss, occurring at 200–400 °C [52, 53].

**Table 5:** Comparative study on glass transition temperature of epoxy-silica composites from DSC reported in literature with the present study.

Silica filler			Curing agent	Maximum Change in T_g_, °C	Reference
Size	Modification	Content, %			
20 nM	Methyl Isobutylketone	1–5	Methyl-hexahydropthalic anhydride	Decreased by 10 °C	[46]
3μm, 10 nm	-	50 phr (3μm) + 1 phr (10 nm)	3 or 4-methy-1,2,3,6-tetrahydropthalic	Increased by 1 °C	[51]
10–20 nm	-	10–70	4,4- diamino-diphenylmethane	Decreased by 12–45 °C	[55]
100 nm	-	5–40	Hexahydro-4-methylpthalic anhydride	Decreased by 30 °C	[56]
23 nm, 74 nm, 170 nm	Organosilane	0–30	Piperidine	No significant effect	[57]
20 nm	diglycidyl ether of bisphenol A	12	3,3- diaminodiphenylsulfone (DDS)	Decreased by 3 °C	[58]
5 μm (mesoporous)	-	3	4,4-diamino diphenylsulfone	Increased by 3.65–5.75 °C	Present study

The temperature of the thermal decomposition was found to be shifted to higher values for all the composites in comparison with the silica-free control, suggesting a synergistic effect of silica fillers on the enhancement of the thermal stability of the cured resin. Such prominent behavior could be explained by the lower surface energy of silica particles, providing thermal insulation to the matrix at high temperature [54].

Table 6 depicts the thermal decomposition of the samples at 10% weight loss, T_d10_, and char residue at 600 °C. Figure 7(b) and Table 7 showed that the composite containing mesoporous micro-particles showed higher thermal stability than those of the nanocomposites and nonporous micro-composites with the highest pore size (EMC-5p50 shows the highest T_d10_, 382 °C). In case of polymer, thermal degradation is associated with the network structure of the polymer and proceeds when the vibrational energy exceeds the primary bonding between the atoms [2]. In the present study, we expected that increasing the pore size of the silica particles would cause the resin to enter into the pores in order to form an interpenetrating network (IPN), increasing the thermal stability. This was assumed to be the driving force for the maximum enhancement of the thermal stability of the composite, EMC-5p50. On the contrary, composites with modified silica showed comparatively lower degradation temperature, attributed to the interrupted bonding of the surface silanol groups with resin caused by the silane coupling agent.

**Table 6:** Comparative study on thermal stability of epoxy-silica composites reported in literature with the present study.

Silica filler			Curing agent	Maximum increment in thermal stability, °C	Reference
Size	Modification	Content, %			
Nanoscale colloidal silica	Methylisobutylketone	0–30	4,4-diaminodiphenylmethane	10	[53]
			Diethylphosphite	9	
10–20 nm	Methylisobutylketone	10–70	4,4-diaminodiphenylmethane	25	[54]
5μm (mesoporous)	-	3	4,4-diaminodiphenylsulfone	16	Present study

**Table 7:** The results of thermal stability of 4,4′-DDS cured epoxy-silica composites at 10 °C/min in N_2_ atmosphere.

Sample name	T_d10_, (°C)	Char residue at 600 °C, (wt %)
CE	366.00	0
ENC-50	375.50	1.66
EMC-3p10	379.00	2.70
EMC-5p20	380.00	1.10
EMC-5p30	381.30	0.30
EMC-5p50	382.00	1.62
EMC-20	376.00	0.45
EMC-w	380.70	0.50

Since the dehydroxylation of the silanol groups takes place at about 600 °C, so char residue at 600 °C was considered [14]. The average char yield at 600 °C was found to increase from 0% for the CE to a maximum of 2.7% for EMC-3p10, as shown in Figure 7(c) and Table 7. However, this increase in char residue was relatively smaller compared with the amount of silica incorporated into the resin. Such behavior indicated that the silica had an insignificant enriching effect on the char formation, as followed by literature [55].

### 3.4 Degradation kinetics analysis

**Figure 8:**
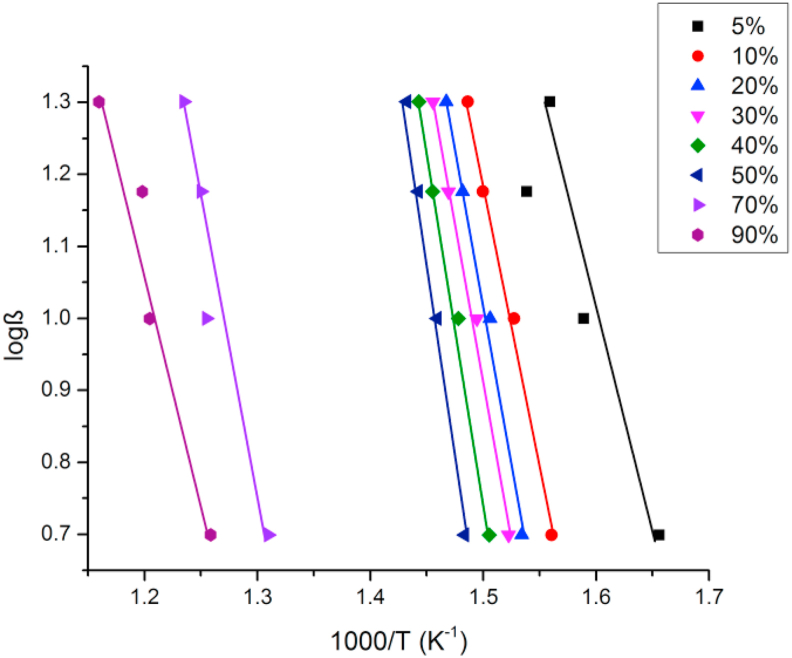
The plots for determination of E_a_ for different degradation % of CE according to FWO method.

**Figure 9:**
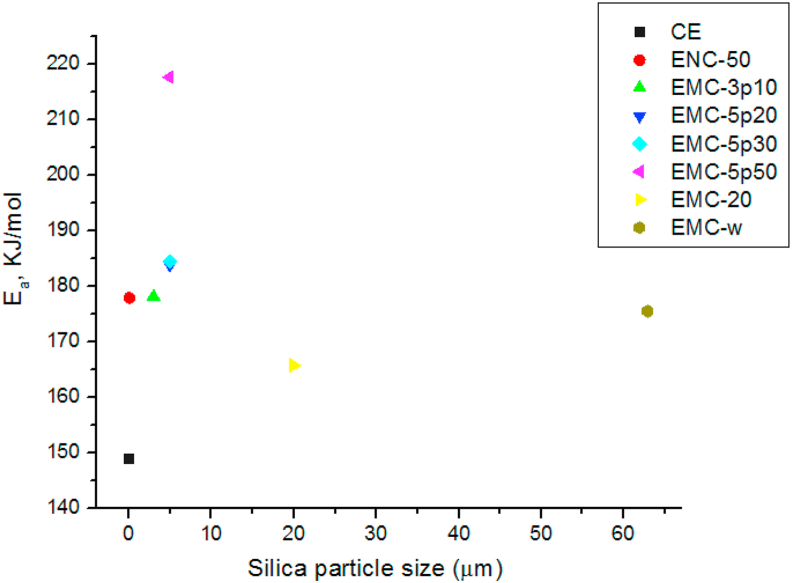
Plot of the average activation energy, E_a_ for thermal degradation of the epoxy-silica composites varying with particle size of silica fillers.

Evaluation of thermal degradation behavior by the FWO method is a practical and convenient way to estimate the shelf-life of the samples. Nonisothermal kinetic analysis of activation energy is pertinent to evaluate the thermal lifetime of polymeric materials without considering the humidity pressure or other service conditions; the service life of a material can be predicted accurately by the equations derived by Toop, utilizing the kinetic parameters calculated from this isoconversional method [59, 60]. Ozawa's method was followed to analyze the kinetics of the thermal degradation of the samples, utilizing TGA curves at different heating rates (5 °C/min, 10 °C/min, 15 °C/min, and 20 °C/min) under an N_2_ atmosphere. An example of plots calculating the activation energy (E_a_) of thermal degradation for different degrees of resin by following the FWO method is shown in Figure 8 (showing good linearity), and the calculated data of E_a_ for the degradation of the composite sample at different percentages of CE, were presented in Table 8. Activation energy (E_a_) data calculated at various percentages of thermal degradation of the composite samples were arranged in combination and plotted, assigning the variation of the average activation energy of composites with the variation of the particle size and pore size in Table 9 and Figure 9, respectively. From Table 9, it is evident that the activation energy gradually increased owing to the heat-resistant property of the silica filler; however, it takes place a non-linear fashion with an increase in the degree of degradation of up to 50%, jumping to lower values with further degradation. It is noteworthy to mention that the activation energy of the neat epoxy for 5% thermal degradation was 86.69 kJ·mol^−1^, whereas higher values were obtained for those of the other silica-filled composites (EMC-5p50 demanded a sufficiently high value of 212.79 kJ·mol^−1^). Similar trends were also found for the mean activation energy for the thermal degradation of the cured epoxies. The mean activation energy for thermal degradation of the neat epoxy was 148.86 kJ·mol^−1^ and mesoporous-microsilica-filled composites showed the maximum range of activation energy, with the highest average value of 217.6 kJ·mol^−1^, demonstrated by EMC-5p50.

**Table 8:** The activation energy E_a_ at different degradation % of CE at N_2_ atmosphere.

Degree of conversion, %	y = mx + c	E_a,_ KJ.mol^−1^	Average E_a_, KJ.mol^−1^
5	y = -4.76x + 8.59	86.690	148.86
10	y = -7.93x + 13.08	144.29	
20	y = -8.80x + 14.29	160.20	
30	y = -8.81x + 14.13	160.42	
40	y = -9.50x + 15.01	172.96	
50	y = -11.67x + 18.02	212.45	
70	y = -7.69x + 10.75	139.99	
90	y = -6.25x + 8.59	113.87

**Table 9:** The activation energy E_a_ calculated at different degradation % of all the composites at N_2_ atmosphere, utilizing FWO method.

Sample name	E_a_ for varying degree of conversion, KJ.mol^−1^								
	5 %	10 %	20 %	30 %	40 %	50 %	70 %	90 %	Average
CE	86.69	144.29	160.20	160.42	172.96	212.45	139.99	113.87	148.86
ENC-50	166.30	186.27	179.10	173.76	188.50	221.30	187.49	117.97	177.84
EMC-3p10	105.37	178.92	179.50	186.06	192.30	270.70	191.80	119.06	178.00
EMC-5p20	100.37	170.33	181.92	189.80	192.87	269.98	223.78	141.95	183.87
EMC-5p30	98.70	163.38	166.17	169.51	190.10	234.51	231.60	141.46	184.44
EMC-5p50	212.79	220.50	223.76	231.56	233.42	273.10	220.73	125.03	217.60
EMC-20	160.93	167.82	163.61	176.34	181.66	206.40	151.26	117.60	165.70
EMC-w	146.01	158.40	166.02	170.41	188.66	232.45	224.10	117.55	175.45

Owing to the complex degradation reaction mechanism of the resin and composites, such analogous behavior of activation energy along with the thermal degradation reaction has been indicated. When incorporated into resin, silica fillers provided a thermal protection layer on the surface of the resins, owing to the low potential surface energy as well as thermal resistance, along with the formation of a barrier to the permeable volatile products produced from the thermal decomposition of the organic moieties present in the resin [52, 53, 55]. More precisely, homogeneously dispersed silica particles into the cured epoxy system leveled up their activation energy required for thermal degradation, attributed to the improved compatibility between the silica fillers and resin owing to the direct chemical bonding of the silanol groups with the oxirane ring. In addition, in the case of the composites with mesoporous micro-silica, the epoxy resin was assumed to be physically confined within the inorganic scaffold through the IPN, leading to the remarkable enhancement of the thermal stability of the EMC-5p50 composite.

### 3.5 Overview of the progress achieved in present study

The aggregation tendency of nanoparticles in epoxy composites is one of the quite known difficulties faced by researchers during the property enrichment process in epoxy composite synthesis, which is usually avoided by the surface treatment and functionalization of nanoparticles [22, 62]. Having discussed all the analysis results, present investigation gave an overview of homogenous dispersion of mesoporous micro-silica in the prepared composites without the need of any surface modifier for the silica fillers. The method for preparation of epoxy-mesoporous microsilica composites via sonication and shear mixing was proved to be simpler but effective to enhance the thermal properties of the composites, where FTIR analyses confirmed the curing of epoxy resin. SEM and FESEM micrographs noticeably demonstrated better interpenetration of epoxy network into the pores of the uniformly distributed silica fillers of regular sizes.

Synergistic effect of only 3 weight% mesoporous silica fillers in epoxy composites on thermal properties was evident when compared with previous studies, as seen in comparative Table 5, 6, and 10. The increasing trend of T_g_ values, enhanced thermal stability and significantly improved thermal degradation kinetic energy attested a strong interfacial interpenetrating polymeric interaction of unmodified micro-silica of increasing nano-scale pores with the epoxy polymer.

**Table 10:** Comparative study on thermal degredation kinetics of epoxy-silica composites reported in literature with the present study following Flynn-Wall-Ozawa Method.

Sample composition	Thermal degradation, %	Average activation energy, E_a_ KJ.mol^−1^	Reference
Urea formaldehyde resin + α-cellulose	75–100	145.00 ± 10	[63]
DGEBA + thiourea + diethylenetriamine	8–20	140.34	[25]
DGEBA + 3-glycidyloxypropyltrimethoxysilane	10–90	209 [calculated by equivalent KAS method]	[52]
DGEBA + silane modified isocyanuric acid triglycidyl ester	10–90	224.00	[61]
DGEBA +4,4′-DDS + mesoporous micro-silica	5–90	217.60	Present study

## 4 Conclusion

Herein, we demonstrated a facile but efficacious and commercially feasible methodology of fabricating epoxy composites through the incorporation of nano-/micro-silica filler with varying nano-scale porosities to compare the effect of silica porosity and particle size on the enhancement of thermal properties. In the present study, micro-silica with nano-pores had been successfully incorporated into epoxy resin without any surface modification and showed competitively better interfacial performances than modified nanosilica fillers, which explored the possibility of improving the epoxy-silica interfacial interaction, by using a smaller amount of micro-silica than that of nano-silica and neglecting the conventional silane coupling modification system of silica fillers. The composites revealed a uniform dispersion along with much improved compatibility between the resin and silica fillers. Furthermore, a synergistic effect of the filler on the enhancement of the thermal durability of the composites for unmodified micro-silica with increasing nano-scale pores was identified. Therefore, by analyzing all the data, we postulated that the formation of an entangled IPN in the epoxy composite on the mesoporous surface of unmodified micro-silica with enhanced thermal properties could have great potential in the application of high-performance polymer with elongated shelf life under high temperature, ensuing the up-to-date trend of sustainable conservation of resources. Thus, the present work will be practically significant for further research on supremacy of unmodified mesoporous micro silica filler over modified nanosilica filler in propagation of interpenetrating polymeric network throughout the polymeric composites with better dispersion and enhanced thermal properties.

## Declarations

### Author contribution statement

Farzana Yeasmin: Performed the experiments; Analyzed and interpreted the data; Contributed reagents, materials, analysis tools or data; Wrote the paper.

Abul K. Mallik: Conceived and designed the experiments; Analyzed and interpreted the data; Contributed reagents, materials, analysis tools or data; Wrote the paper.

Adib H. Chisty, Fataha N. Robel, Md. Shahruzzaman, Papia Haque, Mohammed Mizanur Rahman, Nanami Hano, Makoto Takafuji, Hirotaka Ihara: Analyzed and interpreted the data; Contributed reagents, materials, analysis tools or data.

### Funding statement

This work was supported by the Ministry of Science and Technology, The People's Republic of Bangladesh and the Sakura Science Exchange Program of the Japan Science and Technology Agency (JST).

### Data availability statement

Data included in article/supplementary material/referenced in article.

### Declaration of interests statement

The authors declare no conflict of interest.

### Additional information

No additional information is available for this paper.
